# Optimizing *in vitro* osteoclastogenesis: bone marrow-derived macrophages differentiation and cell density as critical determinants

**DOI:** 10.7717/peerj.20995

**Published:** 2026-03-25

**Authors:** Jie Li, Xinyi Sun, Changqing Yan, Weiwei Zhao, Dandan Liu, Yang Liu, Shuguo Zheng

**Affiliations:** 1 Department of Preventive Dentistry, Peking University School and Hospital of Stomatology, Beijing, China; 2 National Center for Stomatology, Beijing, China; 3 National Clinical Research Center for Oral Diseases, Beijing, China; 4 National Engineering Research Center of Oral Biomaterials and Digital Medical Devices, Beijing, China; 5 Beijing Key Laboratory of Digital Stomatology, Beijing, China; 6 NHC Key Laboratory of Digital Stomatology, Beijing, China; 7 NMPA Key Laboratory for Dental Materials, Beijing, China

**Keywords:** Osteoclastogenesis, Bone marrow-derived monocyte/macrophage (BMMs), Bone marrow-derived macrophages (BMDM), Cell density, Flow cytometry

## Abstract

**Background:**

Osteoclasts are multinucleated cells essential for bone resorption and remodeling. In healthy bone remodeling, osteoclast activity is tightly coupled with osteoblast activity, but this coupling is disrupted in a range of pathological conditions, such as Paget’s disease of bone and delayed healing of fatigue fractures. *In vitro* models of osteoclastogenesis are therefore crucial for studying the mechanisms of osteoclast differentiation and related bone diseases. Optimizing these models is important for advancing research in bone metabolism and therapeutic strategies.

**Methods:**

In this study, we compared three methods for inducing osteoclast differentiation from mouse bone marrow-derived monocyte/macrophage (BMMs). Method 1 involved direct isolation of BMMs, Method 2 differentiated BMMs into bone marrow-derived macrophages (BMDM), and Method 3 incorporated Ficoll-Paque density gradient centrifugation prior to M-CSF-induced BMDM differentiation. For osteoclast differentiation, all three methods employed a complete medium containing 30 ng/mL M-CSF and 50 ng/mL RANKL. After first using TRAP staining, bone resorption assays, F-actin ring staining, quantitative reverse transcription polymerase chain reaction (RT-qPCR), and Western blot to identify the optimal plating density for each method, we then applied the same assays to compare osteoclastogenesis efficiency across the three methods at their optimal densities.

**Results:**

We found that Method 2, which involved differentiating BMMs into BMDM, yielded the highest proportion of live cells and osteoclast precursors, and exhibited the most efficient osteoclast differentiation. The optimal cell density for osteoclastogenesis was 2.8 ∼ 5.6 × 10^4^ cells/cm^2^ for Methods 2 and 3. In contrast, Method 3, despite the additional purification step, did not significantly improve precursor purity compared to Method 2, indicating that the extra purification did not enhance differentiation efficiency.

**Conclusions:**

This study highlights the importance of precursor cell purity and seeding density in osteoclast differentiation. Method 2 (BMMs to BMDM) provides a simplified and effective approach for *in vitro* osteoclastogenesis, optimizing conditions for studying bone resorption and related diseases.

## Introduction

Osteoclasts are multinucleated cells that play a crucial role in bone resorption. These cells are derived from the monocyte/macrophage lineage and fuse in response to stimulation by macrophage colony-stimulating factor (M-CSF) and receptor activator of nuclear factor-κB ligand (RANKL) (Elson et al., 2022; Feng & Teitelbaum, 2013; Teitelbaum, 2000). The interaction of M-CSF with its receptor, CSF1R, and the binding of RANKL to its receptor, RANK, trigger signaling pathways that lead to osteoclast differentiation, fusion, and activation (Boyce, 2013; Boyle, Simonet & Lacey, 2003; Mun, Park & Park-Min, 2020). Osteoclasts secrete acidic environments and proteolytic enzymes that degrade both the inorganic bone mineral and the organic matrix (Boyle, Simonet & Lacey, 2003; Feng & Teitelbaum, 2013). Osteoclasts are essential for bone remodeling, a process in which bone resorption and bone formation are dynamically coupled to maintain skeletal homeostasis, mineral balance, and tissue repair (Durdan, Azaria & Weivoda, 2022; Kim et al., 2020; Xiang et al., 2024). Dysregulation of osteoclast activity is associated with various diseases, including osteoporosis, rheumatoid arthritis, and bone metastasis (Gu et al., 2024; Jones, Glimcher & Aliprantis, 2011; Lane, 2006). Given their important role in both physiology and pathology, osteoclasts have emerged as a major focus for therapeutic intervention.

Given their role in bone resorption, osteoclasts have become important therapeutic targets for diseases characterized by excessive bone loss, such as osteoporosis, Paget’s disease, and rheumatoid arthritis (Takegahara, Kim & Choi, 2024; Tsukazaki et al., 2021). Therapies targeting osteoclasts, such as denosumab, a monoclonal antibody that inhibits RANKL, have been developed for the treatment of osteoporosis and other bone resorption-related conditions (Bone et al., 2017; Curtis et al., 2024). Additionally, drugs like the selective estrogen receptor modulator raloxifene hydrochloride inhibit osteoclast activity in bone (Schmidt et al., 2021). Other approaches include cathepsin K inhibitors such as odanacatib (MK-0822), a selective inhibitor that acts on bone tissue to suppress resorption (Nakamura et al., 2014). However, despite the success of these therapies, challenges remain in obtaining sufficient numbers of osteoclasts for research and clinical applications, particularly when studying osteoclastogenesis and its therapeutic implications in a controlled *in vitro* setting. These challenges arise, in part, from the intrinsic difficulties in studying osteoclasts directly *ex vivo*.

Osteoclasts have traditionally been considered terminally differentiated cells residing primarily within bone tissue, making their isolation in a physiological state challenging. Although recent evidence suggests that RANKL-stimulated osteoclasts may undergo fission into daughter cells (osteomorphs), indicating a potential for cellular recycling, obtaining fully functional osteoclasts directly from bone remains technically difficult (Madel et al., 2018; McDonald et al., 2021). Therefore, reliable and reproducible methods for generating osteoclasts *in vitro* are essential for both research and clinical applications. The generation of osteoclasts *in vitro* is typically performed by isolating BMMs and stimulating them with M-CSF and RANKL (Dong et al., 2024; MacLauchlan et al., 2023; Stegen et al., 2024). However, obtaining osteoclasts from BMMs is often challenging due to the complexity of the differentiation process and the potential for variable outcomes across different protocols. Therefore, optimizing osteoclast differentiation protocols remains a key goal for osteoclast biology research.

Several methods have been proposed for improving the efficiency of osteoclast differentiation, including variations in culture media, cytokine concentrations, and cell densities (Cheng et al., 2022; Song et al., 2019). Despite the availability of these protocols, few systematic studies have compared the effectiveness of different methods for osteoclast differentiation. This is especially critical for generating osteoclasts in sufficient numbers and quality for *in vitro* studies of bone resorption and the development of therapeutic interventions.

In this study, we compare three widely used methods for generating osteoclasts from mouse BMMs. These methods include the traditional approach of isolating BMMs (Cheng et al., 2023), the induction of osteoclasts from BMDM differentiated from BMMs (Dong et al., 2024; Gu et al., 2024), and a method involving Ficoll-Paque density gradient centrifugation to isolate BMMs before osteoclast differentiation (Stegen et al., 2024). The aim of this study is to evaluate the efficiency of each method in terms of the purity of osteoclast precursor cells, the efficiency of osteoclast differentiation, and the functional characteristics of the resulting osteoclasts. By comparing these methods, we hope to identify the most effective approach for generating osteoclasts *in vitro*, which will facilitate the study of osteoclastogenesis and related bone resorption processes.

## Materials & Methods

### Animals

All animal experiments were approved by the Peking University Animal Ethics Committee (Approval number: BDKQ-202504300593). Male C57BL/6 mice (6 weeks old) were obtained from Cyagen Biosciences (Guangzhou, China) and housed under specific pathogen-free (SPF) conditions. A total of 30 male mice were used in this study. The sample size was determined based on preliminary cell yield estimates, with an average isolation efficiency of 3∼5  ×   10^7^ cells per mouse. Mice were maintained in individually ventilated cages (IVC; dimensions: 390  ×   200  ×   160 mm) at a density of five mice/cage. The housing environment was controlled with a 12-hour light/dark cycle, temperature of 22 ± 2 °C, and humidity of 55 ± 10%. All mice were acclimatized for 1 week prior to experimental initiation, with no animals requiring euthanasia during this period. Given the terminal nature of the experimental design, no mice were retained post-experiment. Analgesics or anesthetics were not administered, as they were deemed unnecessary for the procedural workflow.

### Isolation of osteoclast precursor cells

Mice were euthanized *via* cervical dislocation and sterilized with 75% ethanol. The femurs and tibiae were aseptically dissected, and the soft tissues were removed using sterile scissors and forceps. The bones were immediately placed in ice-cold phosphate-buffered saline (PBS) and transferred to a sterile biosafety cabinet. The bones were rinsed 4–5 times with sterile PBS containing 1% penicillin/streptomycin (15140-122; Gibco). Approximately one mm of tissue was trimmed from one end of the bones using sterile scissors, and the bones were then placed into self-made 1.5 mL centrifuge tubes with sleeves containing 50 µL α-minimal essential medium (α-MEM; Gibco, C12571500BT) (see Fig. S1 for schematic). The bones were subjected to centrifugation at 8,000 ×g for 20 s at 4 °C to release the bone marrow cells from the bone cavity. This process was repeated three times to ensure the complete release of the bone marrow cells. The collected bone marrow tissue was pooled into a sterile tube.

### Group 1: red blood cell lysis method (uses red blood cell lysis buffer)

One mL of red blood cell lysis buffer (R7757; Sigma-Aldrich) was added to the bone marrow collected from each mouse, as the collected bone marrow volume was largely consistent across animals using a standardized protocol. The bone marrow was incubated at room temperature for 3 min to remove red blood cells. The reaction was neutralized by adding an equal volume of complete α-MEM supplemented with 10% fetal bovine serum (FBS; Gibco; A5669701). The cell suspension was centrifuged at 200 ×g for 5 min, and the resulting pellet was resuspended in complete α-MEM, supplemented with 10% FBS and 1% penicillin/streptomycin. The cells were plated in 10 cm dishes and incubated overnight at 37 °C in a 5% CO_2_ incubator.

### Group 2: Ficoll-Paque gradient centrifugation (removes red blood cells during the gradient separation)

The bone marrow was filtered through a 70 µm cell strainer (431751; Corning, NY, USA) to prepare a single-cell suspension necessary for effective Ficoll-Paque gradient separation. The cells were centrifuged at 200 ×g for 5 min at room temperature. The cell pellet was resuspended in five mL of α-MEM and then processed for density gradient centrifugation. A 15 mL Falcon tube was prepared with five mL of Ficoll-Paque (17144003-1; Cytiva), and the bone marrow suspension was carefully layered on top of the Ficoll-Paque solution. The tube was centrifuged at 450 ×g for 35 min without brake to allow for separation. After centrifugation, the hazy ring (approximately two mL) located at the top of the Ficoll gradient, which contains mononuclear cells, was carefully collected. These cells were then centrifuged at 200 ×g for 5 min, and the pellet was resuspended in complete α-MEM, supplemented with 10% FBS and 1% penicillin/streptomycin. The cells were seeded in 10 cm culture dishes and incubated overnight at 37 °C in a 5% CO_2_ incubator.

After overnight incubation, non-adherent cells were collected as osteoclast precursors and used for subsequent differentiation assays.

### Osteoclast differentiation (using cells from the above isolation phase)

**Figure 1:**
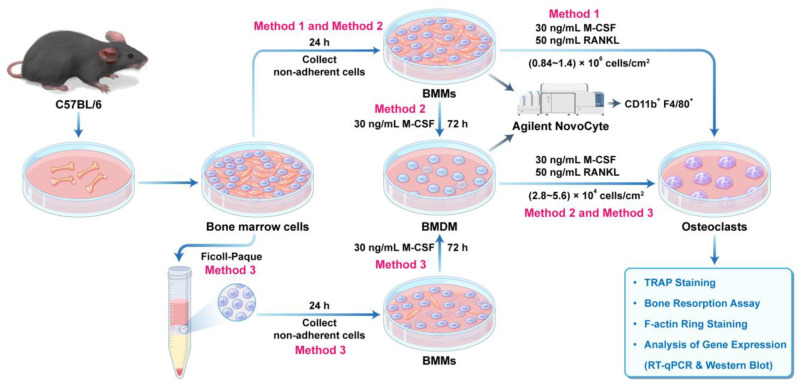
Flow diagram comparing the efficiency of three osteoclast induction methods in bone marrow cells.

Three distinct osteoclast differentiation methods were compared in this study (Fig. 1). All protocols utilized complete α-MEM (supplemented with 10% FBS and 1% penicillin/streptomycin), with cells maintained at 37 °C in a 5% CO_2_ humidified atmosphere. For assays requiring subsequent RNA or protein extraction (*e.g.*, quantitative reverse transcription polymerase chain reaction (RT-qPCR), Western blot), cells were seeded in 12-well plates (1506289; Thermo Fisher Scientific). For assays requiring direct staining and imaging (*e.g.*, TRAP staining, F-actin ring staining), cells were seeded in 24-well plates (142475; Thermo Fisher Scientific). For all protocols, half of the culture medium was replaced with fresh medium every other day during the osteoclast differentiation.

### Method 1: direct differentiation from BMMs (uses cells from Group 1)

The non-adherent osteoclast precursors without undergoing Ficoll-Paque gradient separation, as described earlier, were collected and centrifuged at 112 ×g for 5 min. The supernatant was discarded, and the cells were resuspended in complete medium containing 30 ng/mL recombinant mouse M-CSF (576406; BioLegend) and 50 ng/mL recombinant mouse RANKL (462-TEC-010; R&D Systems). The non-adherent cells were seeded in 24-well or 12-well plates at densities of 0.28  × 10^6^, 0.84  × 10^6^, 1.4  × 10^6^, and 2.0  × 10^6^ cells/cm^2^ for osteoclast differentiation.

### Method 2: macrophage pre-differentiation (uses cells from Group 1)

The non-adherent osteoclast precursors without Ficoll-Paque gradient separation were collected and centrifuged at 112 ×g for 5 min. The supernatant was discarded, and the cells were resuspended in complete medium containing 30 ng/mL recombinant mouse M-CSF. The non-adherent cells were seeded at a density of 2.0  × 10^5^ cells/cm^2^ in 6-well plates (140675; Thermo Fisher Scientific) to induce differentiation of BMDM. After 3 days, the BMDM were treated with StemPro Accutase Cell Dissociation Reagent (A1110501; Thermo Fisher Scientific) to obtain a cell suspension. The BMDM were then seeded in 24-well or 12-well plates at densities of 1.4  × 10^4^, 2.2  × 10^4^, 2.8  × 10^4^, 5.6  × 10^4^, 1.4  × 10^5^, and 2.2  × 10^5^ cells/cm^2^ and cultured in complete medium containing both 30 ng/mL recombinant mouse M-CSF and 50 ng/mL recombinant mouse RANKL for osteoclast differentiation.

### Method 3: macrophage pre-differentiation (Ficoll-Paque gradient separation) (uses cells from Group 2)

Osteoclast precursors undergoing Ficoll-Paque gradient separation were collected and centrifuged at 112 ×g for 5 min. All subsequent procedures, including macrophage induction with M-CSF, cell seeding densities, and differentiation conditions, were identical to those described in Method 2.

### TRAP staining

After 3–4 days of osteoclast induction, the cells were fixed with 4% paraformaldehyde (P1110; Solarbio) at room temperature for 10 min. Following fixation, the cells were washed three times with PBS. The TRAP (Tartrate-resistant acid phosphatase) staining solution was prepared according to the manufacturer’s instructions using the Leukocyte Acid Phosphatase (TRAP) Kit (387A; Sigma-Aldrich). The cells were incubated with the TRAP staining solution at 37 °C in the dark for 40 min. The stained cells were examined under a light microscope (IX53; Olympus). Six fields of 10 × magnification with the highest number of osteoclasts were selected for imaging. The number of TRAP-positive multinucleated cells (≥3 nuclei) was manually counted, whereas the area was quantified using ImageJ software (National Institutes of Health). These cells were considered mature osteoclasts. Three independent biological replicates were performed for each group/experiment.

### Bone resorption assay

BMMs or BMDM were seeded in a bone resorption assay plate (CSR-BRA-24P; Cosmo Bio) to induce osteoclast differentiation. After 7 days of osteoclast induction, the culture medium was discarded, and the cells were removed by soaking in 10% sodium hypochlorite (S101636 NaCIO) for 10 min. The wells were then washed three times with PBS and air-dried. Using a light microscope (IX53; Olypmus), the three largest areas with resorption pits were selected for imaging. The total resorbed area was quantified using ImageJ software, and the percentage of the resorbed area relative to the total culture area was calculated. Three independent biological replicates were performed for each group/experiment.

### F-actin ring staining

After 4 days of osteoclast induction, the culture medium was discarded, and cells were fixed with 4% PFA at room temperature for 10 min. Following fixation, cells were washed three times with PBS. To visualize F-actin structures, cells were incubated with TRITC-phalloidin (CA1610; Solarbio) at a working concentration of 1:200 in PBS for 30 min at room temperature in the dark according to the manufacturer’s instructions. Nuclei were counterstained with Hoechst (C0030; Solarbio) for 5 min at room temperature in the dark. F-actin ring formation was observed using a Leica TCS SP8 confocal microscope (Leica Microsystems). Three independent biological replicates were performed for each group/experiment.

### RNA isolation and quantitative real-time PCR (qPCR)

Total RNA was extracted from differentiated osteoclasts after 4 days of induction using TRIzol Reagent (15596018CN; Invitrogen) according to the manufacturer’s protocol. Genomic DNA (gDNA) contamination was eliminated with DNase I (RNase-free; Accurate Biology; AG12001). RNA concentration was quantified using a NanoDrop Eight spectrophotometer (Thermo Fisher Scientific), yielding concentrations of 500–1,000 ng/µL with A260/A280 ratios ranging from 1.9 to 2.0. Complementary DNA (cDNA) was synthesized from 500 ng of total RNA in a 10 µL reaction volume using PrimeScript RT Master Mix (RR036A; Takara Bio). qPCR was performed on a 7,500 Real-Time PCR System (Applied Biosystems) with FastStart Universal SYBR Green Master (ROX) (4913914001; Roche) under standard thermal cycling conditions (95 °C for 10 min, followed by 40 cycles of 95 °C for 15 s and 60 °C for 1 min). The expression levels of osteoclast differentiation marker genes were normalized to *Rps18* as an endogenous control, and relative quantification was calculated using the 2^−ΔΔCt^ method. All primers were synthesized by Sangon Biotech (Shanghai, China) (Table 1). Three independent biological replicates were performed for each group/experiment.

### Protein extraction and western blot analysis

After 4 days of osteoclast induction, differentiated osteoclasts were lysed in RIPA lysis buffer (HX1863; Huaxingbio) supplemented with 50 × protease inhibitor cocktail (HX1862; Huaxingbio). Protein concentrations were determined using a BCA assay (A55864; Thermo Fisher Scientific). The samples were denatured by heating at 95 °C for 10 min with 5 × loading buffer (HX1760; Huaxingbio). Equal amounts of protein were separated by 4–12% SDS-PAGE (ET12412Gel; ACE Biotechnology) and transferred onto 0.45 µm PVDF membranes (Millipore, USA; IPVH00010). The membranes were blocked with quick blocking buffer (HX3307; Huaxingbio) for 30 min at room temperature and then incubated with primary antibodies overnight at 4 °C with gentle shaking. After washing, the membranes were incubated with HRP-conjugated secondary antibodies (anti-rabbit, 1:20,000; Proteintech; SA00001-2) for 1 h at room temperature. Protein bands were detected using enhanced chemiluminescence (ECL) substrate (P10300; NCM Biotech) and visualized with a chemiluminescent imaging system (Vilber Lourmat, France). Three independent biological replicates were performed for each group/experiment.

**Table 1:** Primer sequences used for RT-qPCR.

**Name**	**Sequence (5′–3′)**
*Trap* Forward	GCGACCATTGTTAGCCACATACG
*Trap* Reward	CGTTGATGTCGCACAGAGGGAT
*Ctsk* Forward	AGCAGAACGGAGGCATTGACTC
*Ctsk* Reward	CCCTCTGCATTTAGCTGCCTTTG
*Mmp9* Forward	GCTGACTACGATAAGGACGGCA
*Mmp9* Reward	TAGTGGTGCAGGCAGAGTAGGA
*Atp6v0d2* Forward	ACGGTGATGTCACAGCAGACGT
*Atp6v0d2* Reward	CTCTGGATAGAGCCTGCCGCA
*Rps18* Forward	CGGAAAATAGCCTTCGCCATCAC
*Rps18* Reward	ATCACTCGCTCCACCTCATCCT

The following primary antibodies were used: Anti-TRAP (1:1,000, ab191406; Abcam), Anti-CTSK (1:1,000, ab187647; Abcam), anti-MMP9 (1:1,000, 24317T), anti-ATP6V0D2 (1:2,000, NBP1–54595; Novus Biologicals), and anti-GAPDH (1:2,000, 5174S; CST).

### Flow cytometry analysis

BMMs or BMDM obtained using the three methods described above were analyzed for the expression of cell surface CD11b and F4/80 antigens prior to osteoclast differentiation using standard flow cytometry techniques. The staining protocol was as follows: Calcein AM-FITC (E-CK-A354; Elabscience), CD11b-PE-Cy7 (Elabscience, USA; E-AB-F1081H), and F4/80-APC (E-AB-F0995E; Elabscience).

The cells were first incubated with an Fc receptor blocking antibody (Purified Anti-Mouse CD16/32, E-AB-F0997A; Elabscience) for 10 min at 4 °C, followed by staining with the appropriate antibodies at the recommended concentrations for 30 min at 4 °C in the dark. Control samples included a blank tube (no antibody), single positive tubes (Calcein AM, CD11b, or F4/80 alone), and a full staining tube (Calcein AM, CD11b, and F4/80). Three biological replicates were performed for each group. Flow cytometry analysis was conducted using an Agilent NovoCyte flow cytometer (Agilent Technologies) to detect the proportion of live cells in each group as well as the expression of CD11b and F4/80 on the surface of osteoclast precursors. Data acquisition and analysis were carried out using FlowJo software v10.8.1 (FlowJo, USA). Data have been deposited in Figshare (DOI: 10.6084/m9.figshare.30058759).

### Statistical analysis

All data are presented as mean ± standard deviation (SD). All acquired data were included in statistical analysis without exclusions. The normality of all datasets was assessed using the Shapiro–Wilk test in SPSS (version 26; IBM Corp., Armonk, NY, USA). Data that satisfied the normality assumption were analyzed by one-way analysis of variance (ANOVA), whereas data that violated this assumption were analyzed using the Kruskal-Wallis test. Both the one-way ANOVA and Kruskal-Wallis tests were performed using GraphPad Prism (version 8.0.1; GraphPad Software, San Diego, CA, USA).

## Results

### Osteoclastogenesis induced by BMMs at higher densities, with optimal induction observed at 0.84 ∼ 1.4  ×  10^**6**^ cells/cm^**2**^

**Figure 2:**
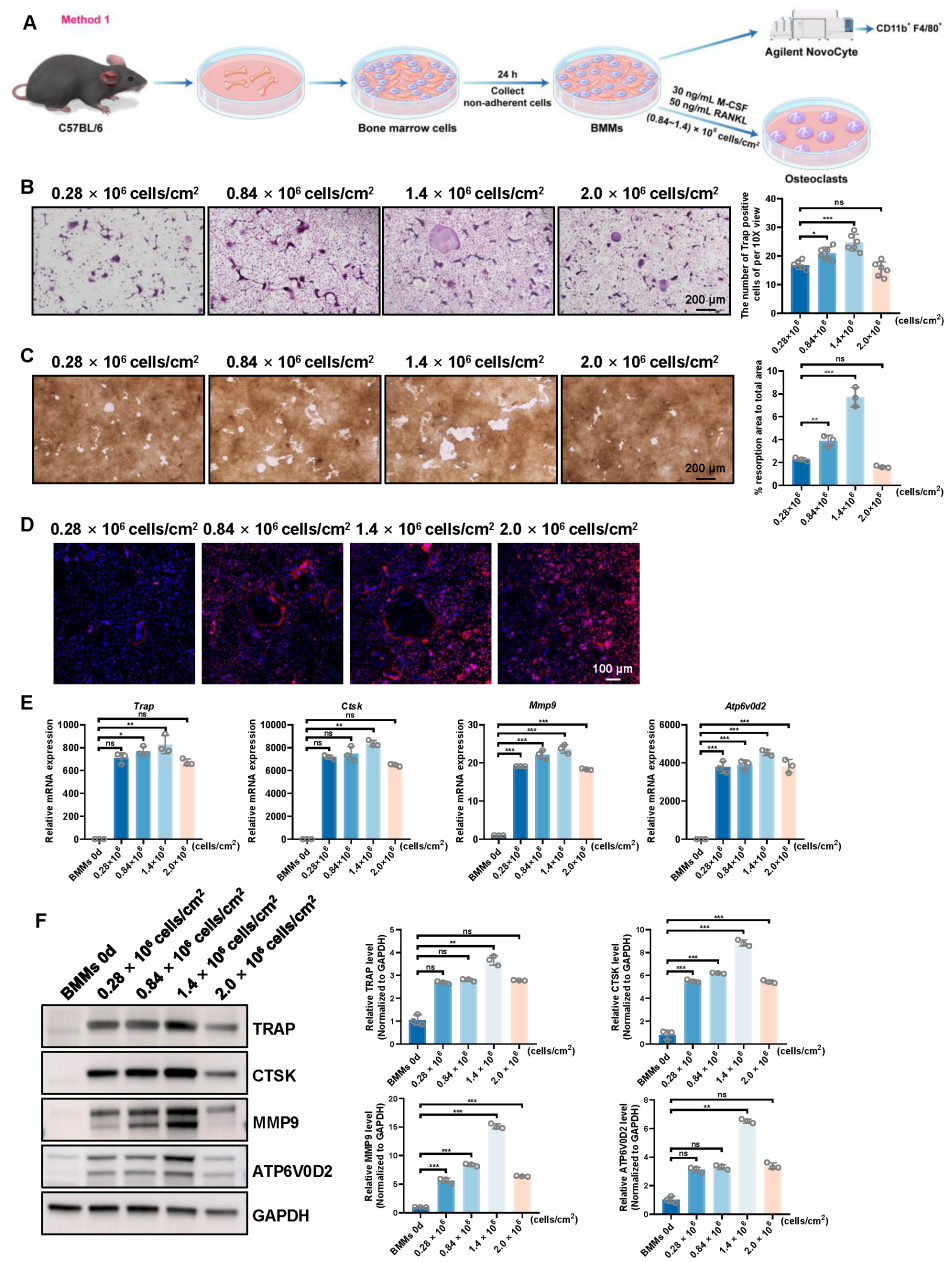
Evaluation of osteoclast-inducing ability using the first bone marrow cell-derived osteoclast induction protocol. (A) Schematic workflow of the osteoclast induction assay. (B) TRAP staining images and quantification of TRAP-positive multinucleated osteoclasts at different plating densities (*n* = 6, 10X views). (C) Representative resorption pit images and quantification of pit area (*n* = 3, 10X views). (D) F-actin ring formation visualized by phalloidin staining. (E) RT-qPCR analysis of osteoclast-related genes normalized to Rps18 (*n* = 3). (F) Western blot analysis and quantitative analysis of osteoclast markers, with GAPDH as a loading control. Data are presented as mean ± SD. Statistical significance for panels (B), (C), (E), and (F) was determined using either one-way ANOVA or the Kruskal-Wallis test, as appropriate. Significance levels: *** *P* < 0.001, ** *P* < 0.01, * *P* < 0.05; ns, not significant (*P* ≥ 0.05).

To determine the optimal conditions for osteoclastogenesis from BMMs, we examined osteoclast differentiation and bone resorption at different cell densities (0.28  × 10^6^, 0.84  × 10^6^, 1.4  × 10^6^, and 2.0  × 10^6^ cells/cm^2^) (Fig. 2A).

Initially, the TRAP staining analysis revealed a progressive increase in the number of TRAP-positive osteoclasts as cell density increased, peaking at 0.84  × 10^6^ cells/cm^2^ and 1.4  × 10^6^ cells/cm^2^ (Fig. 2B and Fig. S2). In contrast, at the lowest density (0.28  × 10^6^ cells/cm^2^), fewer TRAP-positive osteoclasts were observed, and at the highest density (2.0  × 10^6^ cells/cm^2^), there was a slight reduction in TRAP-positive osteoclasts, suggesting a potential inhibitory effect at higher cell concentrations.

Subsequently, bone resorption activity was assessed through the resorption pit assay, where the resorption area relative to the total area was quantified. The resorption activity was markedly higher in the 0.84  × 10^6^ cells/cm^2^ and 1.4  × 10^6^ cells/cm^2^ groups, showing a significant increase in resorption compared to the 0.28  × 10^6^ cells/cm^2^ group (Fig. 2C). However, the 2.0  × 10^6^ cells/cm^2^ group exhibited a decline in resorption (Fig. 2C), further supporting the hypothesis that excessive cell density can impair osteoclast differentiation.

Moreover, F-actin ring formation, a hallmark of mature osteoclasts, was most prominent in the 0.84  × 10^6^ cells/cm^2^ and 1.4  × 10^6^ cells/cm^2^ groups (Fig. 2D). In contrast, these structures were less prominent in the 2.0  × 10^6^ cells/cm^2^ group, aligning with the reduced functionality observed at higher densities.

Gene expression analysis further validated these findings. RT-qPCR (Fig. 2E) and Western blot analysis (Fig. 2F) showed a significant upregulation of osteoclast-related genes, such as *Trap*, *Ctsk*, *Mmp9*, and *Atp6v0d2*, in all groups. The highest expression levels were observed at 0.84  × 10^6^ cells/cm^2^ and 1.4  × 10^6^ cells/cm^2^ groups, with a slight reduction at 0.28  × 10^6^ cells/cm^2^ and 2.0  × 10^6^ cells/cm^2^.

In conclusion, the results consistently demonstrate that osteoclast differentiation and function are most efficiently induced at moderate cell densities (0.84∼1.4  ×10^6^ cells/cm^2^). These densities led to the highest number of TRAP-positive osteoclasts, the greatest resorption activity, and the most organized F-actin rings. In contrast, higher cell densities (2.0  × 10^6^ cells/cm^2^) resulted in a reduction of osteoclast differentiation and function, likely due to a density-dependent inhibitory effect. Therefore, for optimal osteoclastogenesis from BMMs *in vitro*, a cell density of 0.84  × 10^6^ cells/cm^2^ to 1.4  × 10^6^ cells/cm^2^ is recommended.

### Optimized osteoclastogenesis induced by BMDM at low density (2.8∼5.6  × 10^**4**^ cells/cm^**2**^)

**Figure 3:**
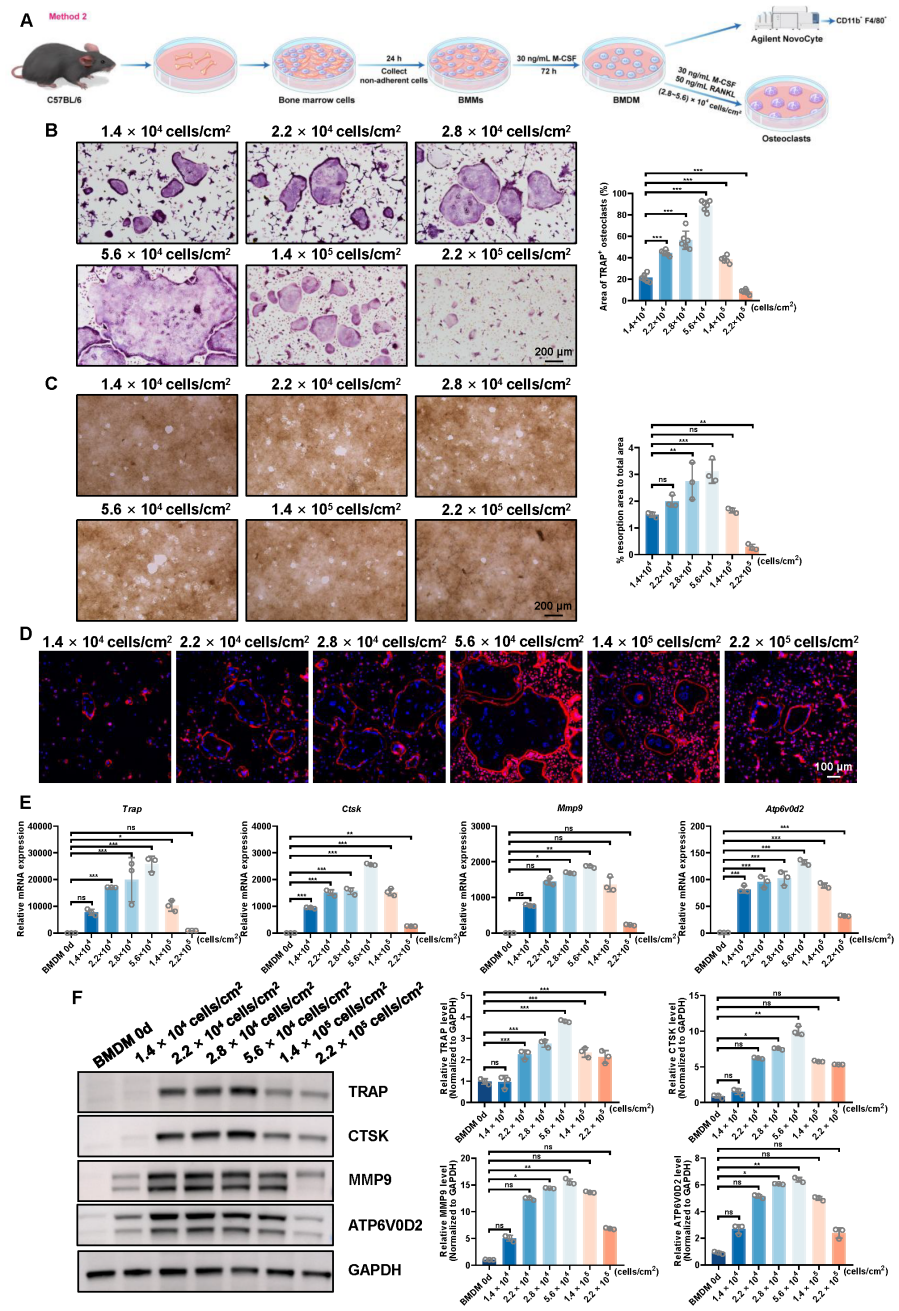
Evaluation of osteoclast-inducing ability using the second bone marrow cell-derived osteoclast induction protocol. (A) Schematic workflow of the osteoclast induction assay. (B) TRAP staining images and quantification of TRAP-positive multinucleated osteoclasts at different plating densities (*n* = 6, 10X views). (C) Representative resorption pit images and quantification of pit area (*n* = 3, 10X views). (D) F-actin ring formation visualized by phalloidin staining. (E) RT-qPCR analysis of osteoclast-related genes normalized to Rps18 (*n* = 3). (F) Western blot analysis and quantitative analysis of osteoclast markers, with GAPDH as a loading control. Data are presented as mean ± SD. Statistical significance for panels (B), (C), (E), and (F) was determined using either one-way ANOVA or the Kruskal-Wallis test, as appropriate. Significance levels: *** *P* < 0.001, ** *P* < 0.01, * *P* < 0.05; ns, not significant (*P* ≥ 0.05).

To identify the optimal conditions for osteoclast differentiation, we employed a two-step induction method in which BMMs were first differentiated into BMDM using M-CSF (30 ng/mL) for 72 h. Subsequently, these BMDM were seeded at different densities (1.4  × 10^4^, 2.2  × 10^4^, 2.8  × 10^4^, 5.6  × 10^4^, 1.4  × 10^5^, and 2.2  × 10^5^ cells/cm^2^) and induced with both M-CSF and RANKL to promote osteoclastogenesis (Fig. 3A).

The TRAP staining results (Fig. 3B and Fig. S3) revealed a density-dependent trend in osteoclast formation. At lower densities (1.4  × 10^4^ and 2.2  × 10^4^ cells/cm^2^), there was limited formation of TRAP-positive osteoclasts, with moderate cell fusion and osteoclasts size. At the 2.8  × 10^4^ and 5.6  × 10^4^ cells/cm^2^ densities, a marked increase in the area of TRAP-positive osteoclasts was observed. However, at higher densities (1.4  × 10^5^ and 2.2  × 10^5^ cells/cm^2^), the area of TRAP-positive osteoclasts decreased, suggesting a density-dependent inhibitory effect at these higher cell concentrations.

The bone resorption pit assay (Fig. 3C) further supported the observed density-dependent effects on osteoclastogenesis. At lower cell densities (1.4  × 10^4^ and 2.2  × 10^4^ cells/cm^2^), the resorption pits were sparse and shallow, indicating limited osteoclast activity. However, at densities of 2.8  × 10^4^ and 5.6  × 10^4^ cells/cm^2^, there was a marked increase in the resorption area, consistent with enhanced osteoclast differentiation observed in TRAP staining assay at these densities. At higher densities (1.4  × 10^5^ and 2.2  × 10^5^ cells/cm^2^), the resorption area decreased, further supporting the inhibitory effect of overcrowding on osteoclast activity.

F-actin ring staining (Fig. 3D) further corroborated these findings. At the lower densities (1.4  × 10^4^ and 2.2  × 10^4^ cells/cm^2^), the F-actin rings were less organized, whereas at 2.8  × 10^4^ and 5.6  × 10^4^ cells/cm^2^, the rings were more prominent and well-formed. The F-actin ring formation diminished at higher densities (1.4  × 10^5^ and 2.2  × 10^5^ cells/cm^2^), consistent with reduced osteoclast functionality at excessive cell densities.

Gene expression analysis confirmed the findings. RT-qPCR (Fig. 3E) and Western blot analysis (Fig. 3F) demonstrated a significant upregulation of osteoclast-related genes, including *Trap*, *Ctsk*, *Mmp9*, and *Atp6v0d2*, in the 2.8  × 10^4^ and 5.6  × 10^4^ cells/cm^2^ groups. The highest expression levels of *Trap* and *Ctsk* were observed at these densities, with minimal expression at the highest density (2.2  × 10^5^ cells/cm^2^). This suggests that moderate cell densities enhance osteoclast differentiation, while excessive density may hinder this process.

In conclusion, the results consistently demonstrate that osteoclast differentiation from BMDM are most efficiently induced at moderate cell densities, specifically between 2.8  × 10^4^ and 5.6  × 10^4^ cells/cm^2^. The data from TRAP staining, bone resorption pit assay, F-actin ring formation, RT-qPCR, and Western blot all converge on these densities as optimal for osteoclastogenesis, while higher densities (1.4  × 10^5^ and 2.2  × 10^5^ cells/cm^2^) impair osteoclast differentiation and functionality, likely due to overcrowding and diminished cell fusion capacity. These findings provide important insights into the optimal conditions for *in vitro* osteoclast differentiation and will guide future experiments in osteoclast biology.

### Osteoclastogenesis induced by BMDM isolated *via* Ficoll-Paque density gradient separation, with optimal induction observed at 2.8∼5.6  × 10^**4**^ cells/cm^**2**^

**Figure 4:**
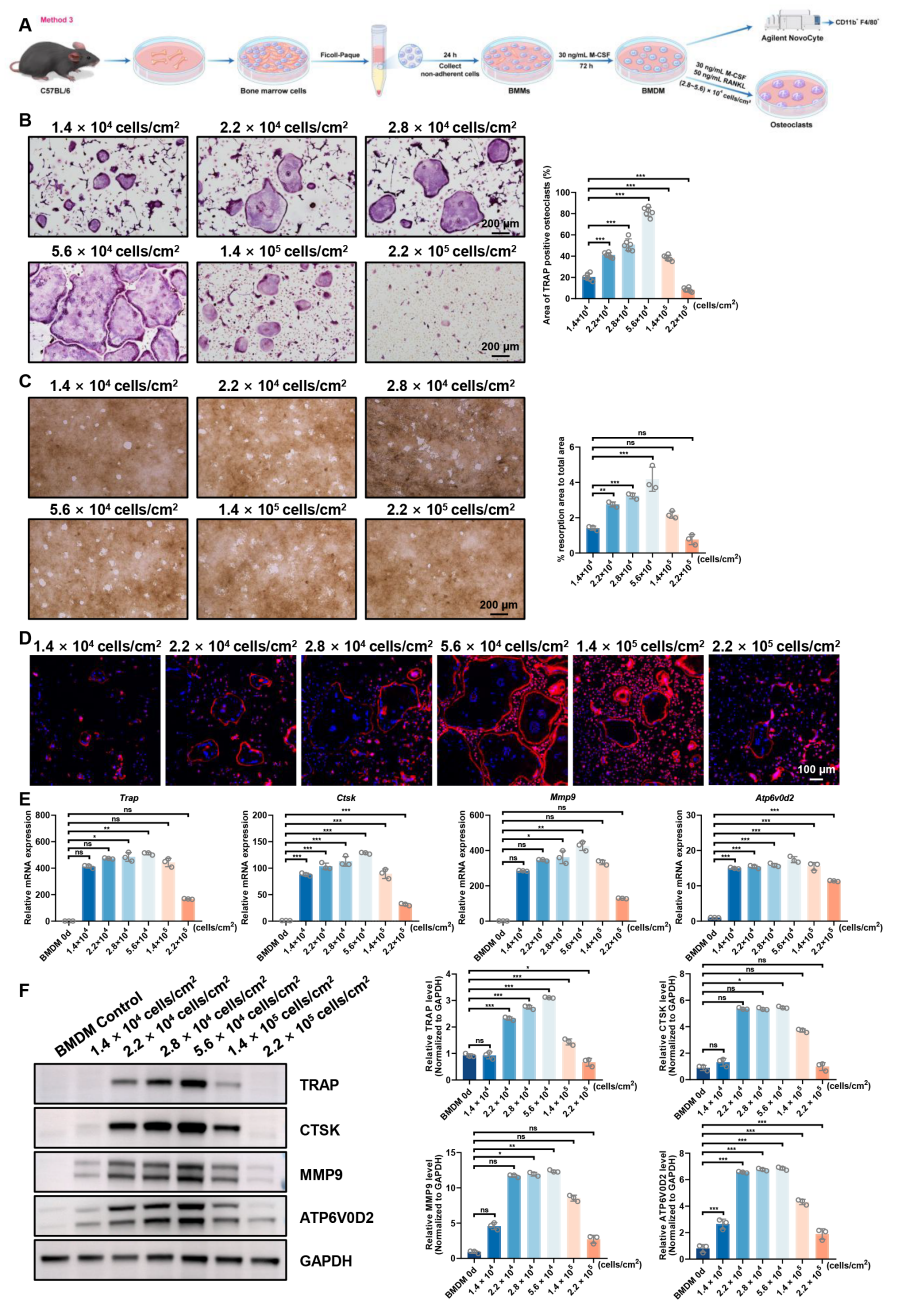
Evaluation of osteoclast-inducing ability using the third bone marrow cell-derived osteoclast induction protocol. (A) Schematic workflow of the osteoclast induction assay. (B) TRAP staining images and quantification of TRAP-positive multinucleated osteoclasts at different plating densities (*n* = 6, 10X views). (C) Representative resorption pit images and quantification of pit area (*n* = 3, 10X views). (D) F-actin ring formation visualized by phalloidin staining. (E) RT-qPCR analysis of osteoclast-related genes normalized to Rps18 (*n* = 3). (F) Western blot analysis and quantitative analysis of osteoclast markers, with GAPDH as a loading control. Data are presented as mean ± SD. Statistical significance for panels (B), (C), (E), and (F) was determined using either one-way ANOVA or the Kruskal-Wallis test, as appropriate. Significance levels: *** *P* < 0.001, ** *P* < 0.01, * *P* < 0.05; ns, not significant (*P* ≥ 0.05).

To refine osteoclast differentiation conditions, we employed a modified two-step induction method for BMDM. In this third method, BMMs were first isolated using Ficoll-Paque density gradient centrifugation to enrich the mononuclear cell fraction. The isolated cells were then differentiated into BMDM over 72 h with M-CSF (30 ng/mL). Subsequently, BMDM were seeded at varying densities (1.4  × 10^4^, 2.2  × 10^4^, 2.8  × 10^4^, 5.6  × 10^4^, 1.4  × 10^5^, and 2.2  × 10^5^ cells/cm^2^) and induced with M-CSF and RANKL to promote osteoclastogenesis, following the same approach as in Method 2 (Fig. 4A).

The results were largely consistent with those of Method 2. TRAP staining demonstrated a clear density-dependent pattern in osteoclast formation (Fig. 4B and Fig. S4). The 2.8∼5.6  × 10^4^ cells/cm^2^ densities exhibited the highest number of TRAP-positive multinucleated cell formation, with enhanced cell fusion efficiency and largest osteoclast cell sizes. In contrast, higher densities (1.4∼2.2  × 10^5^ cells/cm^2^) showed reduced and smaller TRAP-positive osteoclasts. These findings were further supported by bone resorption assays, where 2.8∼5.6  × 10^4^ cells/cm^2^ cultures displayed extensive pit formation compared to markedly decreased resorption areas at 1.4∼2.2  × 10^5^ cells/cm^2^ (Fig. 4C).

Cytoskeletal analysis revealed well-organized F-actin rings at 2.8∼5.6  × 10^4^ cells/cm^2^ that became progressively smaller at higher densities (Fig. 4D). At the molecular level, peak expression of osteoclast-related markers (*Trap*, *Ctsk*, *Mmp9*, and *Atp6v0d2*) was observed at 2.8∼5.6  × 10^4^ cells/cm^2^, with progressive downregulation at increasing densities (Figs. 4E to 4F).

In conclusion, Method 3 which incorporates Ficoll-Paque density gradient centrifugation prior to M-CSF induction, leads to osteoclast differentiation with similar outcomes to Method 2. The optimal osteoclast differentiation and function were achieved at densities of 2.8  × 10^4^ and 5.6  × 10^4^ cells/cm^2^.

### Flow cytometry analysis of osteoclast precursor cells isolated using three different methods

To evaluate the efficiency of different methods for isolating osteoclast precursor cells, we used flow cytometry to assess the expression of CD11b and F4/80, two key markers for identifying macrophage-lineage cells in the bone marrow. Flow cytometry was performed on cells isolated using three different methods, and the results were consistent across biological replicates.

**Figure 5:**
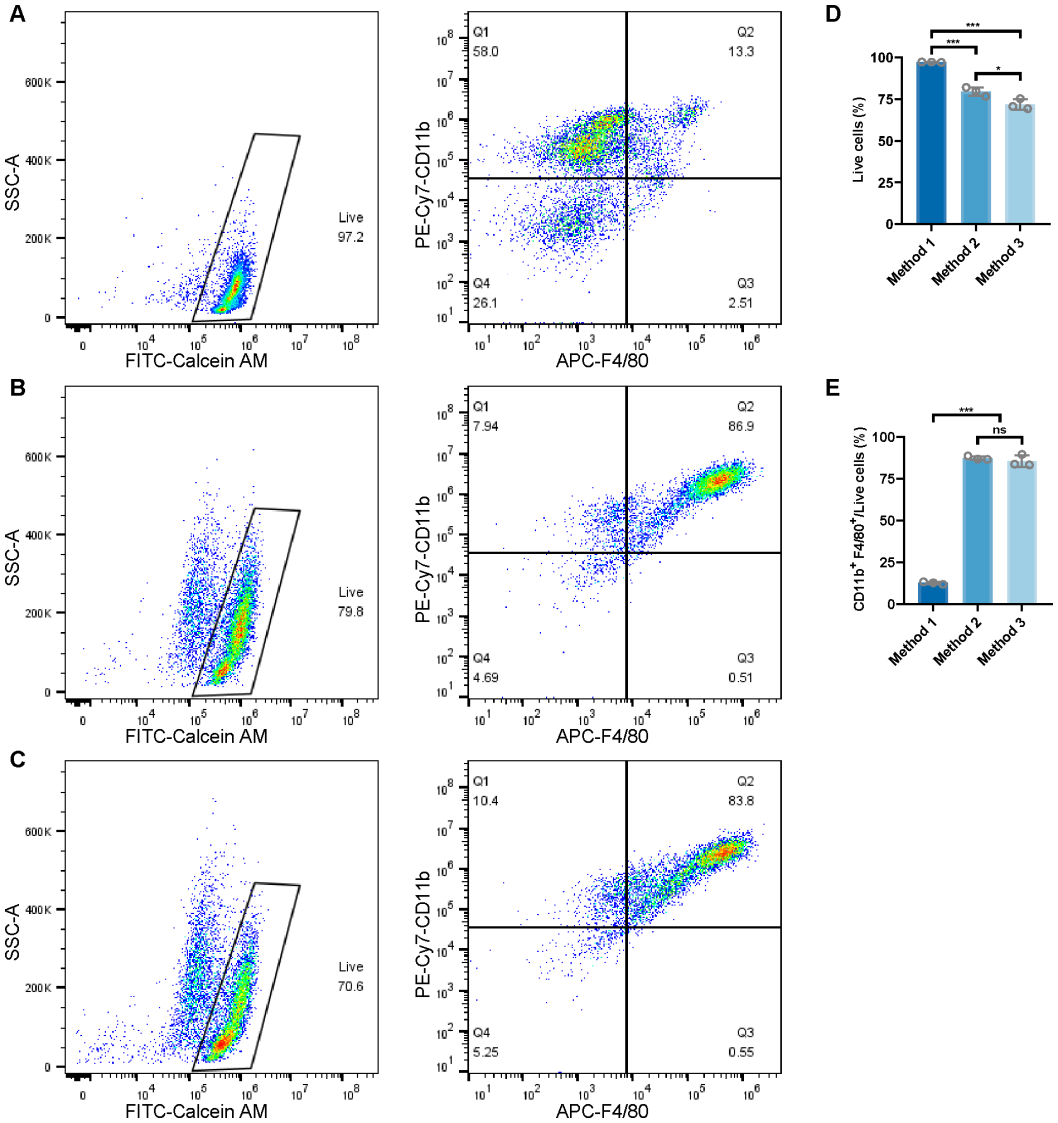
Flow cytometry analysis of osteoclast precursor cells isolated using three different methods. (A) Bone marrow mononuclear cells isolated using a standard protocol. Flow cytometry analysis revealed that 71.3% of the isolated cells were CD11b + mononuclear cells, with low expression of F4/80. (B) Osteoclast precursor cells induced from bone marrow mononuclear cells into macrophages. After induction with M-CSF, the majority of cells (86.9%) were CD11b + F4/80 + macrophages, demonstrating efficient macrophage enrichment for osteoclast differentiation. (C) BMDM isolated using Ficoll -Paque density gradient separation. This method resulted in a population of 83.8% CD11b + F4/80 + macrophages. (D) Proportion of live cells obtained by the three different osteoclast precursor isolation methods (*n* = 3). (E) Proportion of CD11b + F4/80 + macrophages obtained by the three different osteoclast precursor isolation methods (*n* = 3). Data are presented as mean ± SD. Statistical significance for panels (D) and (E) was determined using either one-way ANOVA or the Kruskal-Wallis test, as appropriate. Significance levels: *** *P* < 0.001, ** *P* < 0.01, * *P* < 0.05; ns, not significant (*P* ≥ 0.05).

**Method 1** (Fig. 5A and Fig. S5A): BMMs were isolated using a standard protocol without further purification. Flow cytometry analysis revealed that the majority of the osteoclast precursor cells were CD11b^+^ mononuclear cells, accounting for approximately 70.6% of the isolated population. Compared to the other methods, the F4/80 signal in this population was less prominent, indicating limited macrophage differentiation at this stage.

**Method 2** (Fig. 5B and Fig. S5B): Following isolation, BMMs were differentiated into BMDM using M-CSF. Flow cytometry analysis demonstrated that this approach yielded a highly enriched population of CD11b^+^ F4/80^+^ macrophages, with approximately 87.4% of the cells co-expressing both markers. These results indicate that M-CSF pre-differentiation efficiently generates committed osteoclast precursors. Biological replicates confirmed the consistency of this method, further validating its robustness for enriching CD11b^+^ F4/80^+^ cell population.

**Method 3** (Fig. 5C and Fig. S5C): To assess whether additional purification could enhance macrophage purity, BMMs were first isolated *via* Ficoll-Paque density gradient centrifugation before M-CSF differentiation.

Surprisingly, flow cytometry revealed no significant difference in the proportion of CD11b^+^ F4/80^+^ cells (approximately 85.6%) compared to Method 2 (87.4%) (Figs. 5D to 5E). While Ficoll-Paque separation is widely used for high-purity mononuclear cell isolation (*e.g.*, in peripheral blood studies), its application here did not significantly improve macrophage purity over direct M-CSF differentiation, suggesting that the additional purification step may be redundant for osteoclast precursor isolation from bone marrow.

In summary, flow cytometry analysis showed that Method 2, which involves differentiating BMMs into BMDM, provided the highest proportion of live cells and CD11b^+^ F4/80^+^ osteoclast precursor cells. Despite the incorporation of Ficoll-Paque gradient separation in Method 3, a standard technique for cell purification, it did not enhance macrophage purity compared to Method 2, and the proportion of live cells in the osteoclast precursor population obtained was lower than in Method 2 (Figs. 5D to 5E). These findings underscore that M-CSF-induced differentiation alone (Method 2) is a simpler and more effective strategy for generating osteoclast precursor cells.

### Direct comparison of osteoclast differentiation and bone resorption *via* the three isolation methods

To directly compare the osteoclastogenic capacity of the three methods, we performed a comprehensive analysis at their respective optimal seeding densities (Method 1: 0.84 and 1.4  × 10^6^ cells/cm^2^; Methods 2 & 3: 2.8 and 5.6  × 10^4^ cells/cm^2^).

**Figure 6:**
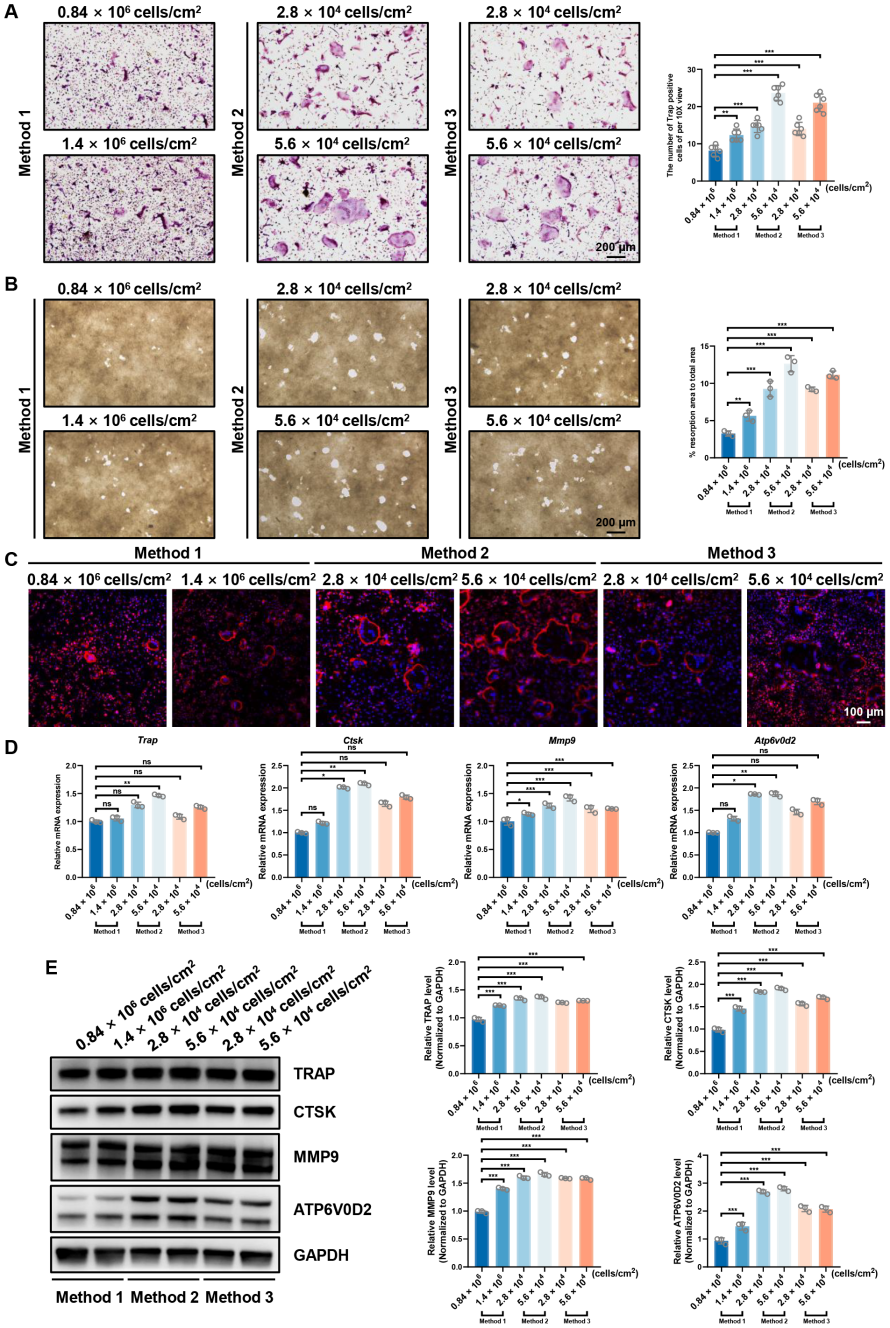
Evaluation of osteoclast-inducing ability using three distinct induction methods at their optimal plating densities. (A) TRAP staining images and quantification of TRAP-positive multinucleated osteoclasts (*n* = 6, 10X views). (B) Representative resorption pit images and quantification of pit area (*n* = 3, 10X views). (C) F-actin ring formation visualized by phalloidin staining. (D) RT-qPCR analysis of osteoclast-related genes normalized to Rps18 (*n* = 3). (E) Western blot analysis and quantitative analysis of osteoclast markers, with GAPDH as a loading control. Data are presented as mean ± SD. Statistical significance for panels (A), (B), (D), and (E) was determined using either one-way ANOVA or the Kruskal- Wallis test, as appropriate. Significance levels: *** *P* < 0.001, ** *P* < 0.01, * *P* < 0.05; ns, not significant (*P* ≥ 0.05).

As shown in Fig. 6A, TRAP staining revealed that Methods 2 and 3 yielded a significantly higher number of TRAP-positive multinucleated cells compared to Method 1. Consistent with this, bone resorption assays (Fig. 6B) demonstrated that osteoclasts derived from Methods 2 and 3 exhibited a markedly greater pit formation area. F-actin ring staining (Fig. 6C) further confirmed robust osteoclast differentiation in these groups, showing more extensive and well-defined sealing zones. The mRNA and protein expression of osteoclast-related genes analyzed by RT-qPCR (Fig. 6D) and Western blot (Fig. 6E), respectively, further corroborated the superior differentiation achieved with Methods 2 and 3. Notably, while Method 3 showed more robust osteoclastogenesis than Method 1, its overall performance across assays was either comparable to or slightly inferior to that of Method 2. Taken together, these direct functional comparisons demonstrate that Method 2 yields osteoclasts with the most potent resorptive activity and differentiation profile among the three methods evaluated.

## Discussion

Osteoclasts are multinucleated cells derived from the monocyte/macrophage lineage and are responsible for the resorption of bone matrix, a crucial process in bone remodeling (Boyce, 2013; Feng & Teitelbaum, 2013). In this study, our systematic comparison of three isolation methods revealed important insights into optimizing *in vitro* osteoclastogenesis models.

Flow cytometry analysis revealed that Method 2, involving M-CSF pre-differentiation of BMMs into BMDM, yielded the highest proportion of live cells (79.5%) and CD11b^+^F4/80^+^ osteoclast precursors (87.4%), indicating effective enrichment of committed precursor cells. This finding aligns with previous studies, which have demonstrated the importance of macrophages in the osteoclast differentiation process (Chandrabalan et al., 2024; Weivoda & Bradley, 2023; Xu et al., 2022). In contrast, Method 3, which incorporated Ficoll-Paque density gradient centrifugation prior to M-CSF pre-differentiation, resulted in a similar proportion of CD11b^+^ F4/80^+^ cells (85.6%), with no significant difference, suggesting that additional purification steps may not significantly improve precursor isolation efficiency for bone marrow-derived cells. This was unexpected, as density gradient centrifugation is commonly used to isolate high-purity macrophage populations in peripheral blood studies (Jia et al., 2018; Meital et al., 2019). The slight decrease in purity observed in this study may be attributed to the fact that additional purification steps do not always lead to higher purity of bone marrow macrophages (Juopperi et al., 2007; Pösel et al., 2012).

The osteoclast differentiation efficiency was most strongly influenced by plating density rather than initial precursor purity. Across Method 2 and 3, optimal differentiation consistently occurred at 1–2  × 10^5^ cells/mL, as evidenced by multiple indicators including robust TRAP-positive multinucleated cell formation, extensive bone resorption activity, well-organized F-actin rings, and upregulation of osteoclast-specific markers (*Trap*, *Ctsk*, *Mmp9*, and *Atp6v0d2*). These observations confirm that cell density is a critical parameter for successful osteoclastogenesis, likely due to its effects on cell–cell interactions and signaling required for proper differentiation and fusion (Wan et al., 2025; Whitlock et al., 2023). It is well-established that the optimal plating density is critical for osteoclastogenesis, as high cell densities may lead to overcrowding and decreased cell fusion, while low densities may not provide sufficient interaction between precursor cells to induce full differentiation. These findings are consistent with previous studies that have highlighted the significance of plating density in osteoclast differentiation (Cheng et al., 2022; Remmers et al., 2023).

These findings have important practical implications for osteoclast research. Method 2 represents a simplified and cost-effective approach for generating functional osteoclasts, which could facilitate studies of bone-related pathologies and therapeutic development. However, there are still some limitations that should be addressed in future studies. While this study used BMMs as the starting material, it would be valuable to compare these methods using other sources of precursor cells, such as peripheral blood mononuclear cells, to assess the generalizability of the findings across different cell types. Furthermore, the translational relevance of our optimized *in vitro* protocol could be evaluated in future studies using appropriate animal models of bone remodeling or osteolytic diseases (Wang, Chen & Wei, 2022). In addition to cell density, other factors such as the concentrations of M-CSF and RANKL are well-known to influence osteoclastogenesis. In the present study, we employed concentrations of 30 ng/mL M-CSF and 50 ng/mL RANKL, which are within the effective ranges commonly reported in the literature (Cheng et al., 2023; Dong et al., 2024; MacLauchlan et al., 2023) and were found to reliably support precursor survival and differentiation in our experimental setup. The optimal combination may vary depending on the specific source of precursor cells, culture duration, and desired functional readouts. Therefore, future systematic investigations into the interplay between cytokine concentration, cell density, and other culture parameters could further refine differentiation efficiency and functional relevance. Moreover, future studies should also consider more comprehensive molecular profiling of osteoclast differentiation, including the analysis of signaling pathways involved in osteoclastogenesis, such as NF-κB, RANKL-RANK signaling, and the MAPK pathway (Cheng et al., 2023; Lee et al., 2016; Xing, Schwarz & Boyce, 2005), as well as the potential influences of various culture conditions, including extracellular matrix components and cytokine combinations, on osteoclastogenesis merit further exploration (Kitaura et al., 2020; Li et al., 2018; Xiong et al., 2011; Zhou, Zheng & Zhao, 2022).

In conclusion, this study demonstrates that M-CSF-mediated pre-differentiation of BMMs effectively enriches osteoclast precursors, while plating density emerges as the predominant factor determining differentiation efficiency. These findings provide valuable guidance for establishing robust *in vitro* models of osteoclastogenesis, with potential applications in both basic research and drug discovery for bone-related disorders. The methodological insights gained from this comparative study may contribute to more standardized and reproducible approaches in osteoclast research.

## Conclusions

In this study, we systematically compared three distinct protocols for inducing osteoclastogenesis from mouse bone marrow-derived monocyte/macrophage lineage cells. Our findings demonstrate that M-CSF-mediated pre-differentiation of bone marrow-derived monocytes/macrophages (Method 2) produces the highest yield of CD11b^+^ F4/80^+^ osteoclast precursors, without requiring additional purification steps such as Ficoll-Paque gradient centrifugation. Although Method 3 also achieved similar differentiation efficiency, the extra purification step did not confer a significant advantage in precursor enrichment.

Importantly, we identified cell seeding density as the most critical determinant of osteoclast differentiation and function across all methods. Optimal osteoclastogenesis was consistently observed at 2.8∼5.6  × 10^4^ cells/cm^2^ for Methods 2 and 3, and at 0.84∼1.4  × 10^6^ cells/cm^2^ for Method 1. These densities supported maximal TRAP-positive cell formation, bone resorption activity, F-actin ring organization, and expression of osteoclast-specific genes.

Collectively, these results provide a practical and reproducible strategy for optimizing *in vitro* osteoclast differentiation. This work not only refines osteoclast induction protocols but also offers methodological insights that will enhance the standardization and reliability of future studies in osteoclast biology and bone disease research.

##  Supplemental Information

10.7717/peerj.20995/supp-1 Supplemental Information 1 Schematic of the self-made centrifuge tubes with sleeves Illustrates the assembly used for sample processing via centrifugation. Key components are labeled as follows: (a) 1.5 mL microcentrifuge tube (sample tube), (b) 0.6 mL microcentrifuge tube (serving as a fitted sleeve), and (c) 16G needle (tube perforated for sample transfer during centrifugation).

10.7717/peerj.20995/supp-2 Supplemental Information 2 Representative TRAP-positive multinucleated osteoclasts after 3 days of osteoclast induction at plating densities from 0.28 × 10^6^ to 2.0 × 10^6^ cells/cm^2^ (method 1)

10.7717/peerj.20995/supp-3 Supplemental Information 3 Representative TRAP-positive multinucleated osteoclasts after 3 days of osteoclast induction at plating densities from 1.4 × 10^4^ cells/cm10^2^ to 2.8 × 10^5^ cells/cm^2^ (method 2)

10.7717/peerj.20995/supp-4 Supplemental Information 4 Representative TRAP-positive multinucleated osteoclasts after 3 days of osteoclast induction at plating densities from 1.4 × 10^4^ cells/cm^2^ to 2.8 × 10^5^ cells/cm^2^ (method 3)

10.7717/peerj.20995/supp-5 Supplemental Information 5 Flow cytometry analysis of osteoclast precursor cells isolated using three different methods (A) Bone marrow mononuclear cells isolated using a standard protocol. (B) Osteoclast precursor cells induced from bone marrow mononuclear cells into macrophages. (C) BMDM isolated using Ficoll-Paque density gradient separation.

10.7717/peerj.20995/supp-6 Supplemental Information 6 Raw data of TRAP staining, bone resorption assay, RT-qPCR and flow cytometry analysis

10.7717/peerj.20995/supp-7 Supplemental Information 7 MIQE checklist

10.7717/peerj.20995/supp-8 Supplemental Information 8 Author Checklist Full

10.7717/peerj.20995/supp-9 Supplemental Information 9 Uncropped blot image for Figure 2F

10.7717/peerj.20995/supp-10 Supplemental Information 10 Uncropped blot image for Figure 3F

10.7717/peerj.20995/supp-11 Supplemental Information 11 Uncropped blot image for Figure 4F

10.7717/peerj.20995/supp-12 Supplemental Information 12 Uncropped blot image for Figure 6E

## Additional Information and Declarations

### Data Availability

The following information was supplied regarding data availability: The data is available at Figshare: Li, Jie (2025). Flow Cytometry Data. figshare. Dataset. https://doi.org/10.6084/m9.figshare.30058759.v1. The raw measurements are available in the Supplemental Files.

## References

[ref-1] Bone HG, Wagman RB, Brandi ML, Brown JP, Chapurlat R, Cummings SR, Czerwiński E, Fahrleitner-Pammer A, Kendler DL, Lippuner K, Reginster JY, Roux C, Malouf J, Bradley MN, Daizadeh NS, Wang A, Dakin P, Pannacciulli N, Dempster DW, Papapoulos S (2017). 10 years of denosumab treatment in postmenopausal women with osteoporosis: results from the phase 3 randomised FREEDOM trial and open-label extension. The Lancet Diabetes & Endocrinology.

[ref-2] Boyce BF (2013). Advances in the regulation of osteoclasts and osteoclast functions. Journal of Dental Research.

[ref-3] Boyle WJ, Simonet WS, Lacey DL (2003). Osteoclast differentiation and activation. Nature.

[ref-4] Chandrabalan S, Dang L, Hansen U, Timmen M, Wehmeyer C, Stange R, Beißbarth T, Binder C, Bleckmann A, Menck K (2024). A novel method to efficiently differentiate human osteoclasts from blood-derived monocytes. Biological Procedures Online.

[ref-5] Cheng Y, Liu H, Li J, Ma Y, Song C, Wang Y, Li P, Chen Y, Zhang Z (2022). Evaluation of culture conditions for osteoclastogenesis in RAW264.7 cells. PLOS ONE.

[ref-6] Cheng HM, Xing M, Zhou YP, Zhang W, Liu Z, Li L, Zheng Z, Ma Y, Li P, Liu X, Li P, Xu X (2023). HSP90*β* promotes osteoclastogenesis by dual-activation of cholesterol synthesis and NF-κB signaling. Cell Death and Differentiation.

[ref-7] Curtis JR, Arora T, Liu Y, Lin TC, Spangler L, Brunetti VC, Stad RK, McDermott M, Bradbury BD, Kim M (2024). Comparative effectiveness of denosumab *vs* alendronate among postmenopausal women with osteoporosis. Journal of Bone and Mineral Research.

[ref-8] Dong Y, Kang H, Peng R, Liu Z, Liao F, Hu SA, Ding W, Wang P, Yang P, Zhu M, Wang S, Wu M, Ye D, Gan X, Li F, Song K (2024). A clinical-stage Nrf2 activator suppresses osteoclast differentiation *via* the iron-ornithine axis. Cell Metabolism.

[ref-9] Durdan MM, Azaria RD, Weivoda MM (2022). Novel insights into the coupling of osteoclasts and resorption to bone formation. Seminars in Cell and Developmental Biology.

[ref-10] Elson A, Anuj A, Barnea-Zohar M, Reuven N (2022). The origins and formation of bone-resorbing osteoclasts. Bone.

[ref-11] Feng X, Teitelbaum SL (2013). Osteoclasts: new insights. Bone Research.

[ref-12] Gu C, Chen P, Tian H, Yang Y, Huang Z, Yan H, Tang C, Xiang J, Shangguan L, Pan K, Chen P, Huang Y, Liu Z, Tang R, Fan S, Lin X (2024). Targeting initial tumour-osteoclast spatiotemporal interaction to prevent bone metastasis. Nature Nanotechnology.

[ref-13] Jia Y, Xu H, Li Y, Wei C, Guo R, Wang F, Wu Y, Liu J, Jia J, Yan J, Qi X, Li Y, Gao X (2018). A modified ficoll-paque gradient method for isolating mononuclear cells from the peripheral and umbilical cord blood of humans for biobanks and clinical laboratories. Biopreservation and Biobanking.

[ref-14] Jones D, Glimcher LH, Aliprantis AO (2011). Osteoimmunology at the nexus of arthritis, osteoporosis, cancer, and infection. Journal of Clinical Investigation.

[ref-15] Juopperi TA, Schuler W, Yuan X, Collector MI, Dang CV, Sharkis SJ (2007). Isolation of bone marrow-derived stem cells using density-gradient separation. Experimental Hematology.

[ref-16] Kim JM, Lin C, Stavre Z, Greenblatt MB, Shim JH (2020). Osteoblast-osteoclast communication and bone homeostasis. Cells.

[ref-17] Kitaura H, Marahleh A, Ohori F, Noguchi T, Shen WR, Qi J, Nara Y, Pramusita A, Kinjo R, Mizoguchi I (2020). Osteocyte-related cytokines regulate osteoclast formation and bone resorption. International Journal of Molecular Sciences.

[ref-18] Lane NE (2006). Epidemiology, etiology, and diagnosis of osteoporosis. American Journal of Obstetrics and Gynecology.

[ref-19] Lee K, Chung YH, Ahn H, Kim H, Rho J, Jeong D (2016). Selective regulation of MAPK signaling mediates RANKL-dependent osteoclast differentiation. International Journal of Biological Sciences.

[ref-20] Li M, Chen X, Yan J, Zhou L, Wang Y, He F, Lin J, Zhu C, Pan G, Yu J, Pei M, Yang H, Liu T (2018). Inhibition of osteoclastogenesis by stem cell-derived extracellular matrix through modulation of intracellular reactive oxygen species. Acta Biomaterialia.

[ref-21] MacLauchlan S, Kushwaha P, Tai A, Chen S, Manning C, Swarnkar G, Abu-Amer Y, Fitzgerald KA, Sharma S, Gravallese EM (2023). STING-dependent interferon signatures restrict osteoclast differentiation and bone loss in mice. Proceedings of the National Academy of Sciences of the United States of America.

[ref-22] Madel MB, Ibáñez L, Rouleau M, Wakkach A, Blin-Wakkach C (2018). A novel reliable and efficient procedure for purification of mature osteoclasts allowing functional assays in mouse cells. Frontiers in Immunology.

[ref-23] McDonald MM, Khoo WH, Ng PY, Xiao Y, Zamerli J, Thatcher P, Kyaw W, Pathmanandavel K, Grootveld AK, Moran I, Butt D, Nguyen A, Corr A, Warren S, Biro M, Butterfield NC, Guilfoyle SE, Komla-Ebri D, Dack MRG, Dewhurst HF, Logan JG, Li Y, Mohanty ST, Byrne N, Terry RL, Simic MK, Chai R, Quinn JMW, Youlten SE, Pettitt JA, Abi-Hanna D, Jain R, Weninger W, Lundberg M, Sun S, Ebetino FH, Timpson P, Lee WM, Baldock PA, Rogers MJ, Brink R, Williams GR, Bassett JHD, Kemp JP, Pavlos NJ, Croucher PI, Phan TG (2021). Osteoclasts recycle *via* osteomorphs during RANKL-stimulated bone resorption. Cell.

[ref-24] Meital LT, Coward AS, Windsor MT, Bailey TG, Kuballa A, Russell FD (2019). A simple and effective method for the isolation and culture of human monocytes from small volumes of peripheral blood. Journal of Immunological Methods.

[ref-25] Mun SH, Park PSU, Park-Min KH (2020). The M-CSF receptor in osteoclasts and beyond. Experimental & Molecular Medicine.

[ref-26] Nakamura T, Shiraki M, Fukunaga M, Tomomitsu T, Santora AC, Tsai R, Fujimoto G, Nakagomi M, Tsubouchi H, Rosenberg E, Uchida S (2014). Effect of the cathepsin K inhibitor odanacatib administered once weekly on bone mineral density in Japanese patients with osteoporosis—a double-blind, randomized, dose-finding study. Osteoporosis International.

[ref-27] Pösel C, Möller K, Fröhlich W, Schulz I, Boltze J, Wagner DC (2012). Density gradient centrifugation compromises bone marrow mononuclear cell yield. PLOS ONE.

[ref-28] Remmers SJA, Van der Heijden FC, Ito K, Hofmann S (2023). The effects of seeding density and osteoclastic supplement concentration on osteoclastic differentiation and resorption. Bone Reports.

[ref-29] Schmidt PJ, Wei SM, Martinez PE, Dor RRB, Guerrieri GM, Palladino PP, Harsh VL, Li HJ, Wakim P, Nieman LK, Rubinow DR (2021). The short-term effects of estradiol, raloxifene, and a phytoestrogen in women with perimenopausal depression. Menopause.

[ref-30] Song C, Yang X, Lei Y, Zhang Z, Smith W, Yan J, Kong L (2019). Evaluation of efficacy on RANKL induced osteoclast from RAW264.7 cells. Journal of Cellular Physiology.

[ref-31] Stegen S, Moermans K, Stockmans I, Thienpont B, Carmeliet G (2024). The serine synthesis pathway drives osteoclast differentiation through epigenetic regulation of NFATc1 expression. Nature Metabolism.

[ref-32] Takegahara N, Kim H, Choi Y (2024). Unraveling the intricacies of osteoclast differentiation and maturation: insight into novel therapeutic strategies for bone-destructive diseases. Experimental & Molecular Medicine.

[ref-33] Teitelbaum SL (2000). Bone resorption by osteoclasts. Science.

[ref-34] Tsukazaki H, Kikuta J, Ao T, Morimoto A, Fukuda C, Tsuda E, Minoshima M, Kikuchi K, Kaito T, Ishii M (2021). Anti-Siglec-15 antibody suppresses bone resorption by inhibiting osteoclast multinucleation without attenuating bone formation. Bone.

[ref-35] Wan Y, Nemoto YL, Oikawa T, Takano K, Fujiwara TK, Tsujita K, Itoh T (2025). Mechanical control of osteoclast fusion by membrane-cortex attachment and BAR proteins. Journal of Cell Biology.

[ref-36] Wang Y, Chen Y, Wei Y (2022). Osteoarthritis animal models for biomaterial-assisted osteochondral regeneration. Biomaterials Translational.

[ref-37] Weivoda MM, Bradley EW (2023). Macrophages and bone remodeling. Journal of Bone and Mineral Research.

[ref-38] Whitlock JM, Leikina E, Melikov K, De Castro LF, Mattijssen S, Maraia RJ, Collins MT, Chernomordik LV (2023). Cell surface-bound La protein regulates the cell fusion stage of osteoclastogenesis. Nature Communications.

[ref-39] Xiang Q, Li L, Ji W, Gawlitta D, Walboomers XF, Van den Beucken J (2024). Beyond resorption: osteoclasts as drivers of bone formation. Cell regeneration.

[ref-40] Xing L, Schwarz EM, Boyce BF (2005). Osteoclast precursors, RANKL/RANK, and immunology. Immunological Reviews.

[ref-41] Xiong J, Onal M, Jilka RL, Weinstein RS, Manolagas SC, O’Brien CA (2011). Matrix-embedded cells control osteoclast formation. Nature Medicine.

[ref-42] Xu H, Zhang S, Sathe AA, Jin Z, Guan J, Sun W, Xing C, Zhang H, Yan B (2022). CCR2+ macrophages promote orthodontic tooth movement and alveolar bone remodeling. Frontiers in Immunology.

[ref-43] Zhou P, Zheng T, Zhao B (2022). Cytokine-mediated immunomodulation of osteoclastogenesis. Bone.

