# Supplementary figures and images for: Optimizing in vitro osteoclastogenesis: bone marrow-derived macrophages differentiation and cell density as critical determinants
Source: PeerJ. 2026 Mar 25;14:e20995. doi: 10.7717/peerj.20995 (PMC13032752; doi:10.7717/peerj.20995)

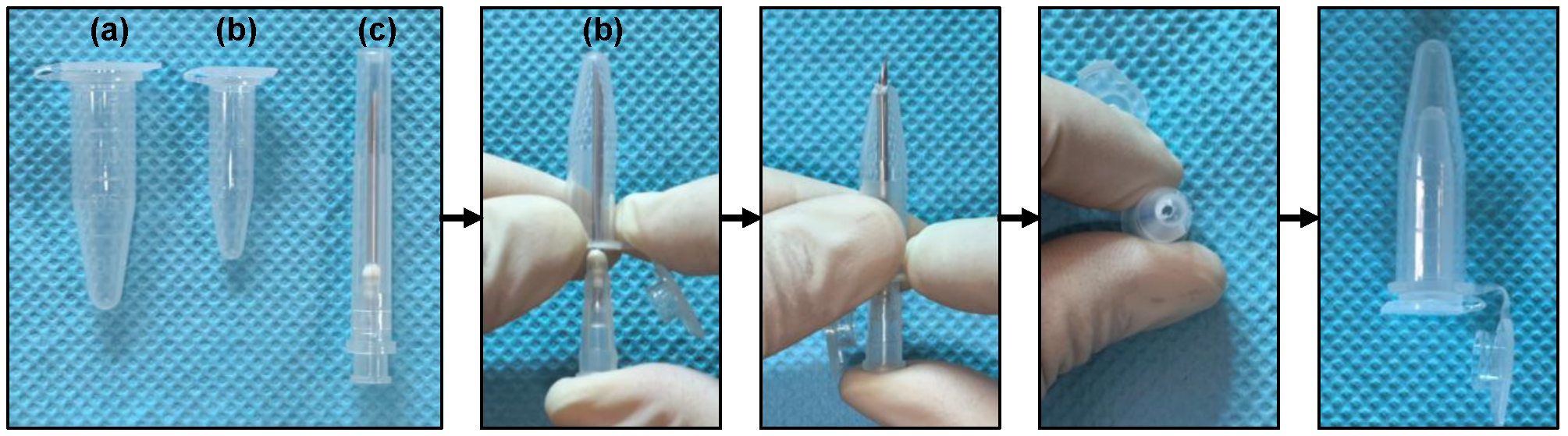

Supplement: Supplemental Information 1 — Illustrates the assembly used for sample processing via centrifugation. Key components are labeled as follows: (a) 1.5 mL microcentrifuge tube (sample tube), (b) 0.6 mL microcentrifuge tube (serving as a fitted sleeve), and (c) 16G needle (tube perforated for sample transfer during centrifugation). [file peerj-14-20995-s001.png]

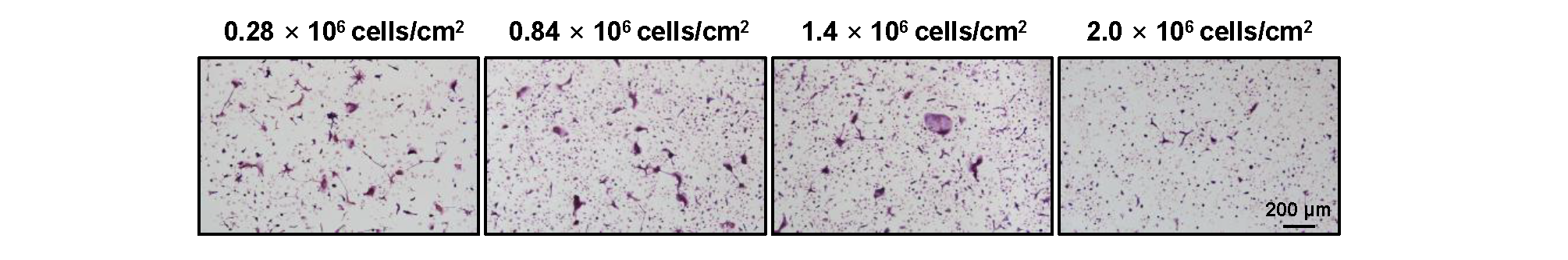

Supplement: Supplemental Information 2 [file peerj-14-20995-s002.png]

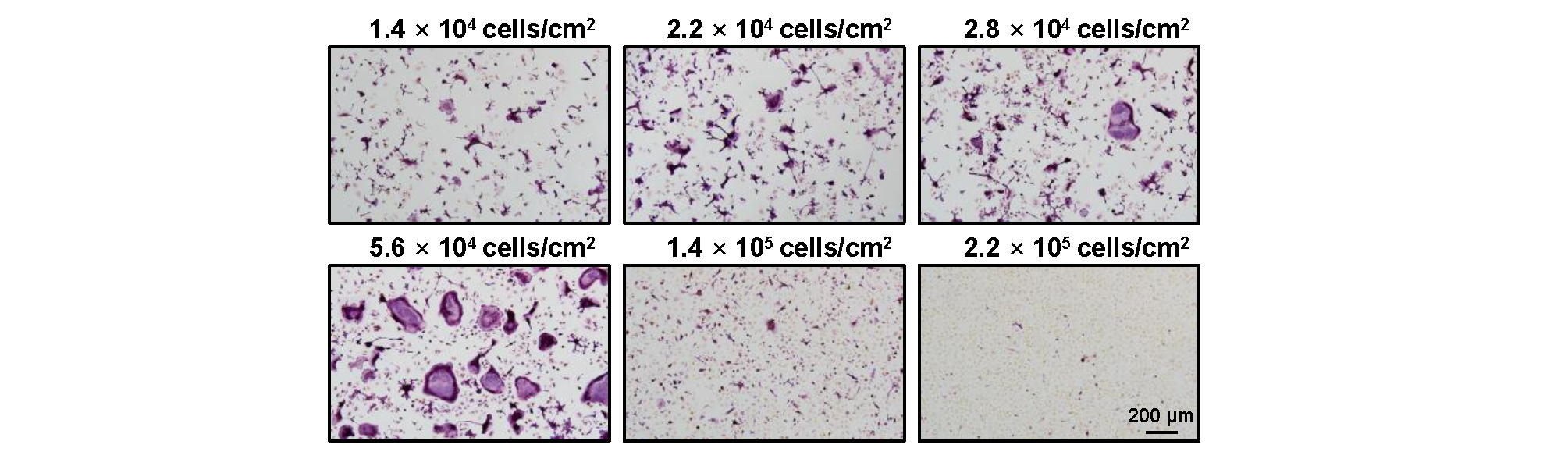

Supplement: Supplemental Information 3 [file peerj-14-20995-s003.png]

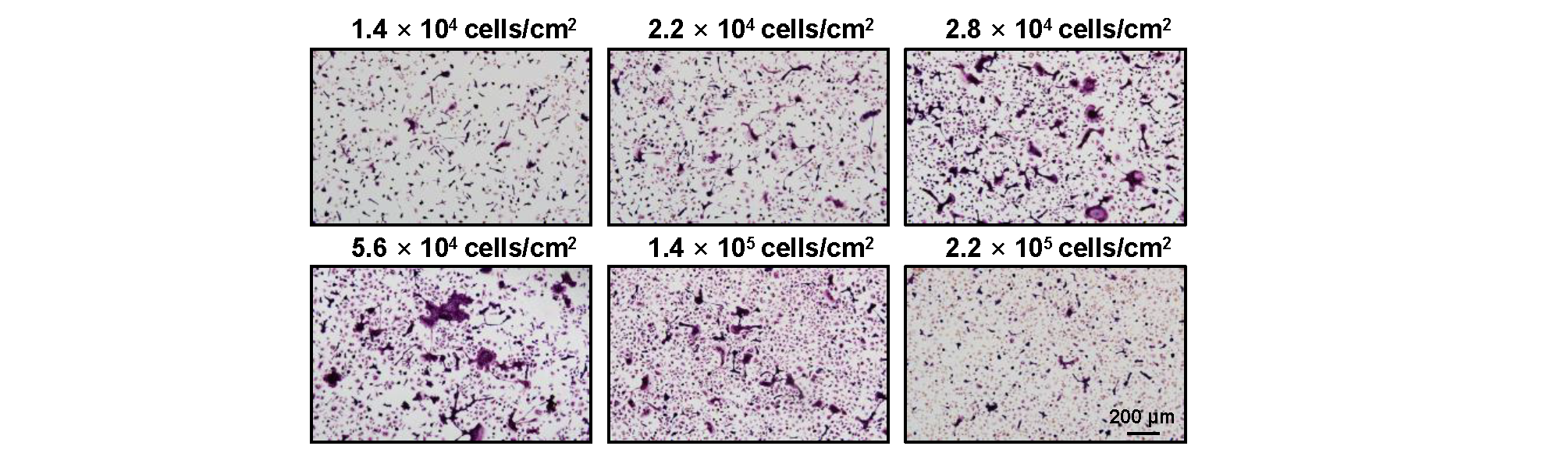

Supplement: Supplemental Information 4 [file peerj-14-20995-s004.png]

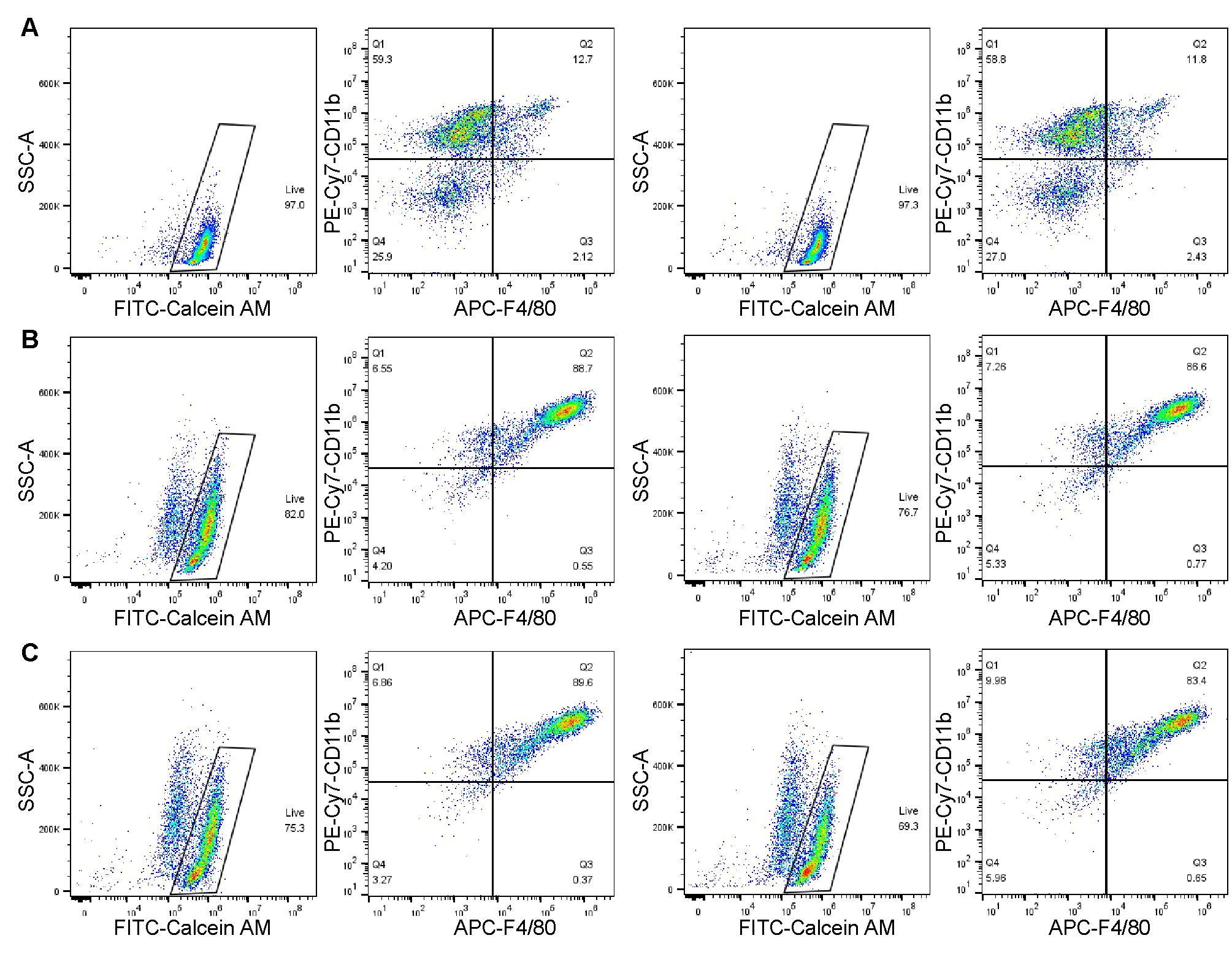

Supplement: Supplemental Information 5 — (A) Bone marrow mononuclear cells isolated using a standard protocol. (B) Osteoclast precursor cells induced from bone marrow mononuclear cells into macrophages. (C) BMDM isolated using Ficoll-Paque density gradient separation. [file peerj-14-20995-s005.png]
