# Supplementary material for: Optimizing in vitro osteoclastogenesis: bone marrow-derived macrophages differentiation and cell density as critical determinants
Source: PeerJ. 2026 Mar 25;14:e20995. doi: 10.7717/peerj.20995 (PMC13032752; doi:10.7717/peerj.20995)
Supplement: Supplemental Information 12 [file peerj-14-20995-s012.pdf]

Uncropped blot image for Figure 6E

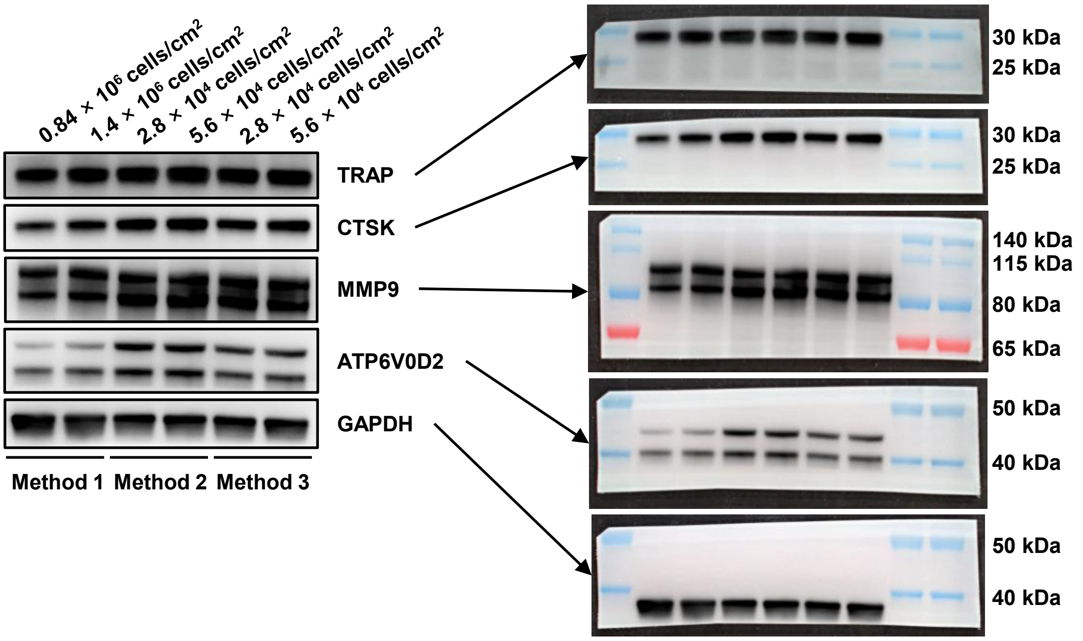

Full-length blot

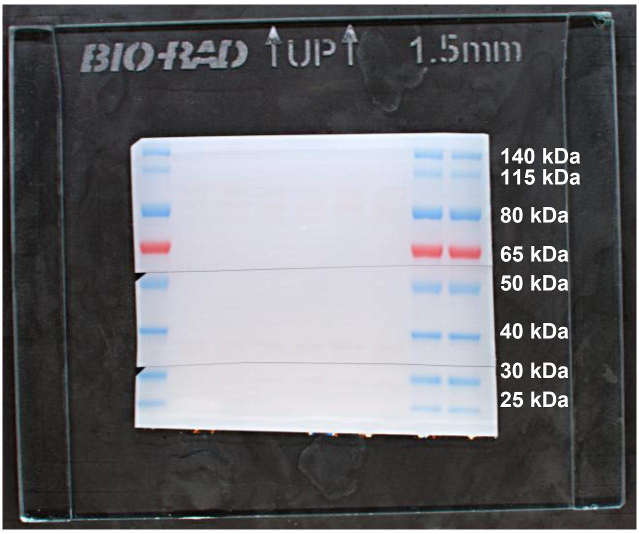

Top blot: MMP9

Medium blot: ATP6V0D2, GAPDH

Bottom blot: TRAP, CTSK

Replicate 2: Uncropped blot image for Figure 6E (replicate 1 shown in Figure 6E)

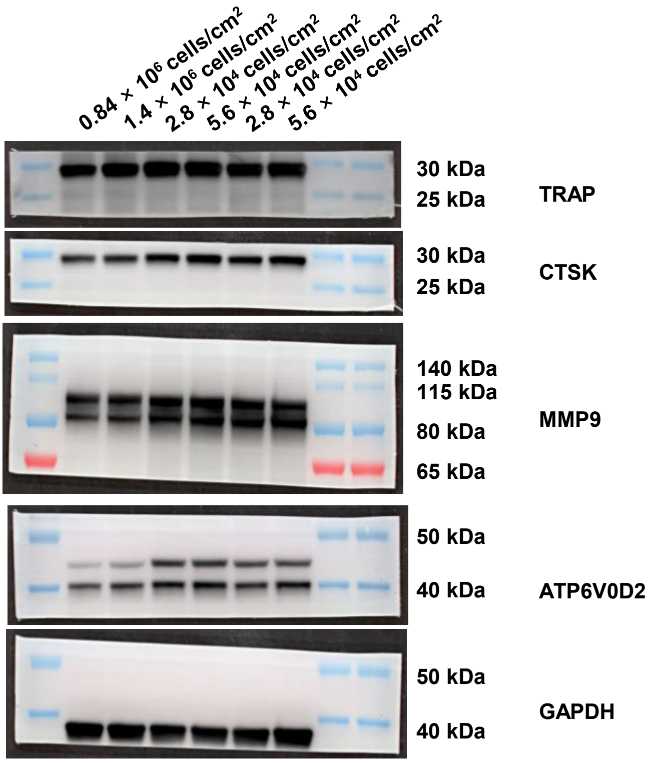

Full-length blot

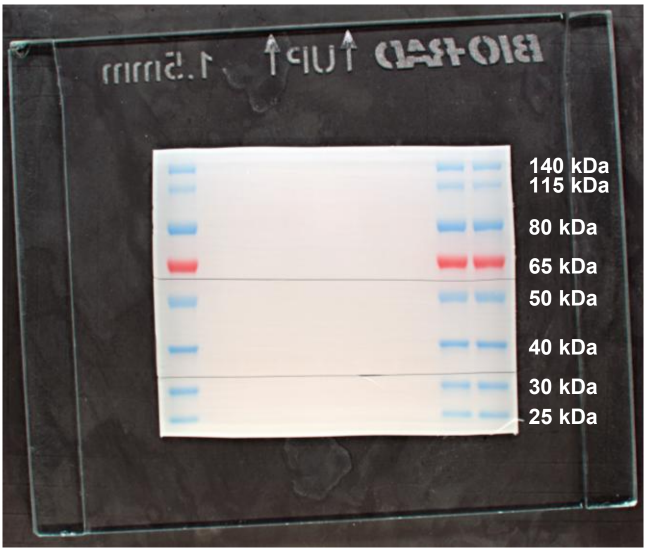

Top blot: MMP9

Medium blot: ATP6V0D2, GAPDH

Bottom blot: TRAP, CTSK
